# Geometric classification of scalp hair for valid drug testing, 6 more reliable than 8 hair curl groups

**DOI:** 10.1371/journal.pone.0172834

**Published:** 2017-06-01

**Authors:** K. Mkentane, J. C. Van Wyk, N. Sishi, F. Gumedze, M. Ngoepe, L. M. Davids, N. P. Khumalo

**Affiliations:** 1 Hair and Skin Research Lab, Division of Dermatology, Groote Schuur Hospital, Cape Town, South Africa; 2 Department of Human Biology, Faculty of Health Sciences, University of Cape Town, Cape Town, South Africa; 3 Statistical Sciences, Faculty of Science, University of Cape Town, Cape Town, South Africa; 4 Engineering, University of Cape Town, Cape Town, South Africa; Northwestern University Feinberg School of Medicine, UNITED STATES

## Abstract

**Introduction:**

Curly hair is reported to contain higher lipid content than straight hair, which may influence incorporation of lipid soluble drugs. The use of race to describe hair curl variation (Asian, Caucasian and African) is unscientific yet common in medical literature (including reports of drug levels in hair). This study investigated the reliability of a geometric classification of hair (based on 3 measurements: the curve diameter, curl index and number of waves).

**Materials and methods:**

After ethical approval and informed consent, proximal virgin (6cm) hair sampled from the vertex of scalp in 48 healthy volunteers were evaluated. Three raters each scored hairs from 48 volunteers at two occasions each for the 8 and 6-group classifications. One rater applied the 6-group classification to 80 additional volunteers in order to further confirm the reliability of this system. The Kappa statistic was used to assess intra and inter rater agreement.

**Results:**

Each rater classified 480 hairs on each occasion. No rater classified any volunteer’s 10 hairs into the same group; the most frequently occurring group was used for analysis. The inter-rater agreement was poor for the 8-groups (k = 0.418) but improved for the 6-groups (k = 0.671). The intra-rater agreement also improved (k = 0.444 to 0.648 versus 0.599 to 0.836) for 6-groups; that for the one evaluator for all volunteers was good (k = 0.754).

**Conclusions:**

Although small, this is the first study to test the reliability of a geometric classification. The 6-group method is more reliable. However, a digital classification system is likely to reduce operator error. A reliable objective classification of human hair curl is long overdue, particularly with the increasing use of hair as a testing substrate for treatment compliance in Medicine.

## Introduction

The variation in hair curvature is the most distinct characteristic of human scalp hair. Studies have indicated that differences in hair curl arise from the bulb [1–3]. Using computer-aided reconstruction of scalp biopsy samples, Lindelof demonstrated that hair emerged from follicles at a right angle to the surface of the scalp for Asian hair and at an acute angle for African hair resulting in straight and curly hair respectively [3]. Recent studies using mouse models show that the size and shape of the dermal papilla dictates hair shaft morphology and thickness [4]. Natural hair curl varies widely across the world possibly due to diversity arising from heredity and environmental factors [5]. However, the use of race (African, European, Asian) as a descriptor of hair form has limitations because it is subjective [6] and presents with overlaps [7,8]. Further, racial admixture makes it difficult to use self-identified race a marker of biological differences.

The accurate identification of hair curl is crucial for forensic investigators wanting to link trace evidence found at crime scenes to suspects [9–11]. Further, certain hair disorders such as folliculitis keloidalis nuchea are predominantly associated with tightly curly hair [12]. Objective characterization of hair would also be useful for hair-curl-specific hair product development. This would better address the needs of different hair curl phenotypes; for example, sebum is not evenly distributed along curly hair shafts resulting in dryer hair [13]. The proposal to abandon the use of race in describing human hair phenotypes [6,14] is even more relevant for the exploding interest in the use of hair as a testing substrate in medicine [15–20]. There is evidence to suggest that curly hair has a higher internal lipid content; [21] this may influence the incorporation of lipid soluble drugs requiring adjustments for hair curl when interpreting results for monitoring treatment compliance in patients such as those infected with Human Immunodeficiency Virus^20^.

A few objective hair classification systems have been attempted [22–24]. The first was introduced by anthropologist Hrdy [23] who assessed hair form variation in seven populations (Bougainville, East Africa, Northwest European, Malaita, Sioux, Japan and Ifugao). He measured the average diameter, medullation, scale count, kinking, average curvature, ratio of maximum to minimum curvature, crimp and ratio of natural to straight hair length then compared within and between population variations using F-tests. The average curvature and ratio of curvature were the two most important variables in describing hair form. Correlation matrices showed a strong correlation between curling variables (kinking, average curvature, ratio of maximum to minimum curvature, crimp and ratio of natural to straight length) while a weak correlation was observed between scale count and the curling variables. Principal component analysis (PCA) showed that 3 components (size and regularity of curl) accounted for 80% of the variance in the data. [23]. Hrdy also observed that the average curvature varied between groups and suggested it would have the greatest contribution in differentiating groups [23]. The precision of the contribution of average curvature to variation was later investigated by Bailey and Schliebe [22] who measured hair curvature of 30 strands (6cm long) from the same individual three times on a template of circles of known radii (and compared means) in one experiment. They described average curvature as an inverse of the radius as did Hrdy [23]. In a second experiment, they compared the curvature of hair form recorded for five family members and found overlaps in recorded means [22]. They did not investigate the reliability by calculating intra and inter rater agreement.

Most recently, De La Mettri expanded on the Bailey & Schliebe system by introducing 3 additional variables (for curly hair), also measured on a 6cm length of proximal hair. They classified hair collected from 1442 volunteers, originating from 18 countries [25], by measuring the curve diameter (CD), the ratio between relaxed and extended hair length (termed the curl index or *i*), the number of twists (*t*) and waves (*w*). The number of waves was counted by constricting a 5cm segment of the hair to 4cm, while the number of twists is the number of natural constrictions along the hair axis [25]. They reported a direct correlation between the number of twists and number of waves, which supported the exclusion of the number of twists measurement for future analyses. Using the remaining three variables, PCA and hierarchical ascendant clustering (HAC), they reported that hair could objectively be classified into 8 groups and provided a set of rules for the classification [25].

This exercise resulted in hair classification rules for the 8-group classification. The curve diameter was used to distinguish four groups in the straight hair spectrum based on cut-off values obtained from the PCA and HAC analysis. Curly hair was differentiated based on combinations of curl index (*i*) scores and maximum number of waves. A follow-up study led by Loussouarn [26] included an additional 1007 volunteers and aimed to demonstrate the reproducibility of the geometric classification. They proposed a simplified approach to the classification, where instead of measuring the curl index (*i*), a curl meter which is a circle with a diameter of 0.98cm was used first to see if the 6cm of hair fits completely (hair curl group VII and VIII) or not (hair V and VI) within the circle. Thereafter the 6cm hair is taped down (0.5cm on each side giving rise) to a 5cm segment which is then constricted to 4cm and the number of waves counted. Thus, classification of the curlier hair groups depended on the combination of the curl meter and number of waves [26].

Although the authors had large sample sizes, they only measured 3 hair fibres sampled from different parts of the head for each volunteer. Further, although the classifications in both [25][26] studies were conducted by different scientists, the inter-rater agreement was not calculated. Both studies also did not report comparisons of repeat measurements (intra-rater reliability).

The aim in this study was to evaluate the reliability of the geometric classification and explore whether reliability could be improved.

## Methods

### Participants and samples

Permission to conduct the study was granted by the Human Research Ethics Committee (HREC) of the Faculty of Health Sciences, (HREC REF:328, IRB00001938) at the University of Cape Town. Participants with at least 6cm of virgin hair (and had not used any chemical straightener or dyes for > 7 months) were invited to participate in the study via notices, fliers and University online research notices. After participants gave written informed consent by signing HREC approved consent forms detailing the objectives and sampling approaches of the study, an area of 0.56cm^2^ (using the same sterilized electric shaver) was shaved from the vertex of the scalp and kept in a dry sealed packet. Prior to geometric measurements, the hair was washed using a 1% sodium dodecyl sulphate solution, rinsed with warm water (37 °C) and air-dried for a minimum of 4 hours. Samples were then stored at room temperature until geometric classification measurements.

### Geometric characterization

Three scientists classified 6cm of proximal virgin hair from the same pool of 48 volunteers using published classification templates and rules [26]. Each rater classified 10 hairs at 2 occasions; therefore, each volunteer’s hair was classified sixty times.

Single strands of natural hair were classified using the curve diameter (CD) meter which identified hair groups I to IV. Six centimetre long strands of hair were place on top of the curve diameter template and a glass slide placed on the hair without shifting or altering its natural curl. The hair was then classified as hair curl group I, II, III or IV by superimposing it to the best fit half-circle. For hair with curls too tight to fit in the curve diameter template, both the curl meter [i.e. hair fits completely (VII and VIII) or does not (V and VI) fit within the circle] and the number of waves calculated [up to 3 for V; ≥4 for VI; up to 5 for VII and ≥ 6 for VIII] were used for classification. For the 6 hair curl group classification, samples were geometrically characterised in the same way as described above, except for the exclusion of the wave count measurement. Thus, samples from the four curly hair groups were classified as groups 5 or 6 based on whether or not the strands fit completely inside the curl meter.

The most frequently occurring hair curl group for each set of 10 strands was reported as the representative hair curl group for the volunteer for each rater at occasion 1 and 2.

### Statistics

The inter and intra-rater agreement, reflecting the reliability of the geometric classification systems was assessed using Cohen’s kappa coefficient (StataCorp. 2013. Stata: Release 13. Statistical Software. College Station, TX: StataCorp LP). The Kappa coefficient measures chance corrected agreement between two or more raters and can range between +1 and -1 [27]. A coefficient of 1 denotes perfect agreement, 0 reflects agreement obtained by chance and a negative value shows worse than expected agreement [28]. Kappa values < 0.20 reflect poor agreement and 0.41–0.60 reflect moderate agreement. Kappa values between 0.61–0.80 are regarded as good agreement, while very good agreement is reflected by kappa values ranging from 0.81 to 1.00 [27,29,30].

## Results

Forty eight volunteers (87% female and 13% male) aged 18–55 years from 4 self-identified racial groups (Asian (5–2 Chinese and 3 Indian), Caucasian (14), Mixed (10) and African (19)) were included. Sixty hair strands were classified for each volunteer for the 8 and again for 6-group classification. It took approximately 15 to 30 minutes to classify 10 strands for curly hair groups (V–VIII) and approximately 10 minutes for straighter hair. In all, 2880 hair strands (48 volunteers X 10 strands X 3 raters X 2 occasions) were initially classified. No rater classified any volunteer’s 10 hairs into the same group; hair was classified into at least 2 (and occasionally 3) groups. The most frequently occurring hair curl group of the 10 strands at each assessment time point was chosen as the representative hair group for the volunteer. One rater went on to classify an additional 80 volunteers to compare the 6-group hair classification with the 8-group system.

The inter-rater agreement for the 8-groups, was fair to moderate [29,30], and higher at occasion 2 (k = 0.455, CI; 0.389–0.521) than occasion 1 (k = 0.380, 95% CI; 0.313–0.447) (Table 1). The intra-rater agreement [ranged from k = 0.444 (95% CI; 0.334–0.553) to k = 0.648 (95% CI; 0.525–0.771)] showed the lowest agreement between the three raters for the most extreme groups (I and VIII, k = 0.170 and k = 0.074 respectively) at occasion 1 (Table 2).

**Table 1:** Inter-rater agreement for the 8-group geometric classification: 3 evaluators at occasion 1 and occasion 2.

Hair Curl Group	Number of	Occasion 1		Occasion 2	
	Hair strands at each occasion	Kappa	95% CI	Kappa	95% CI
**I**	60	0.170	0.007–0.334	0.603	0.440–0.766
**II**	240	0.419	0.256–0.583	0.443	0.279–0.606
**III**	300	0.379	0.216–0.543	0.439	0.275–0.602
**IV**	180	0.515	0.352–0678	0.554	0.391–0.717
**V**	150	0.604	0.441–0.768	0.364	0.200–0.527
**VI**	30	0.182	0.018–0.345	0.407	0.244–0.571
**VII**	360	0.396	0.232–0.559	0.453	0.290–0616
**VIII**	120	0.074	0.000–0.237	0.399	0.236–0.562
**Combined**	1440	0.380	0.313–0.447	0.455	0.389–0.521

**Table 2:** Intra-rater agreement for the 8-group geometric classification: 3 evaluators.

	Agreement	Kappa	95% CI
**Rater 1**	58.33%	0.521	0.413–0.629
**Rater 2**	52.08%	0.443	0.334–0.553
**Rater 3**	70.8%	0.648	0.525–0.771

The third step of the classification for curly hair i.e. counting the number of waves (taping the ends down to count the number of waves) was reported by all 3 raters as laborious, the tiny curly hair as difficult to handle and at risk of breakage. As a result, repeat measurements were again performed at two time points by the 3 raters where the third step [the number of waves count (*w*)] was excluded. This resulted in a 6-group classification where the first step categorized hair curl groups 1–4 similarly to I-IV [26] but ended at the second step where the curl meter classified hair as falling outside of the circle (Group 5 equivalent to V and VI) [26] or completely inside (Group 6 equivalent to VII and VIII). The inter-rater (k = 0.613 at occasion 1 and k = 0.729 at occasion 2) (Table 3) and intra-rater agreements [range: k = 0.599 (95% CI: 0.422–0.776) to k = 0.836 (95% CI; 0.691–0.981)] for the 6-group classification were higher than those of the 8-group classification (Table 4).

**Table 3:** Inter-rater agreement for the 6-group geometric classification: 3 evaluators at occasion 1 and occasion 2.

Hair Curl Group	Occasion 1		Occasion 2	
	Kappa	95% CI	Kappa	95% CI
**1**	0.171	0.008–0.335	0.000	0.000–0.149
**2**	0.534	0.371–0.698	0.610	0.447–0.773
**3**	0.578	0.414–0.741	0.645	0.482–0.809
**4**	0.531	0.367–0.694	0.806	0.643–0.970
**5**	0.206	0.045–0.369	0.463	0.299–0.626
**6**	0.883	0.720–1.000	0.913	0.750–1.000
**Combined**	0.613	0.527–0.699	0.729	0.641–0.817

**Table 4:** Intra-rater agreement for the 6-group geometric classification: 3 evaluators.

	Agreement	Kappa	95% CI
**Rater 1**	87.50%	0.836	0.691–0.981
**Rater 2**	70.83%	0.599	0.422–0.776
**Rater 3**	81.25%	0.751	0.601–0.901

**Fig 1:**
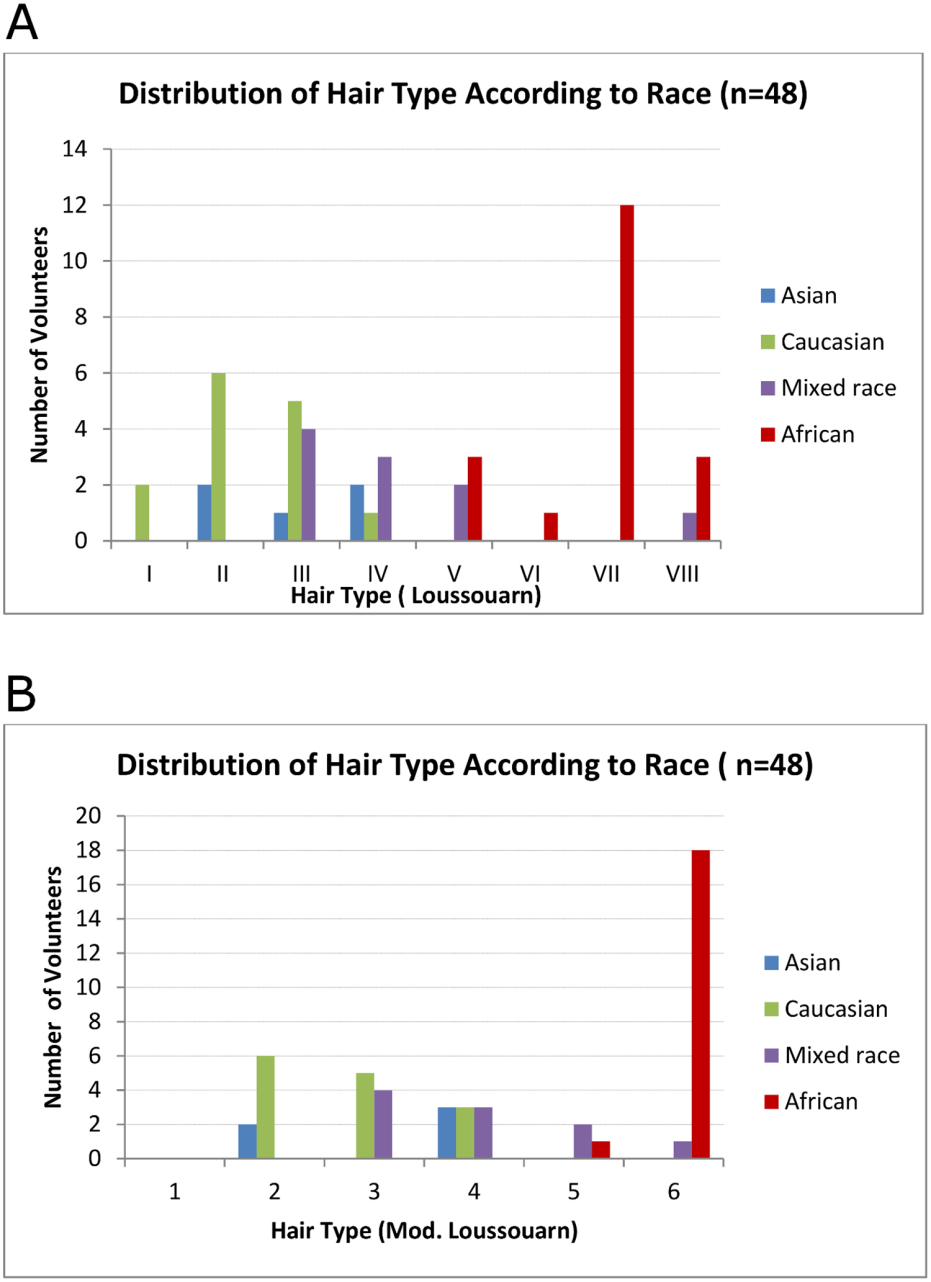
**a. Demographics of sample population (n = 48) of 8 hair curl groups**. Hair geometric measurements done by three assessors and grouped according to self-declared race. Significant overlap observed across groups, confirming the futility of racial hair classification. ‘African hair’ varied from curl group V to VIII. **b. Demographics of sample population (n = 48) of 6 hair curl groups**. Hair geometric measurements done by three assessors and grouped according to self-declared race. The 6-group classification omits the third step (counting the number of waves) and simply classifies high curvature hair groups as falling in/out of the curl meter.

**Fig 2:**
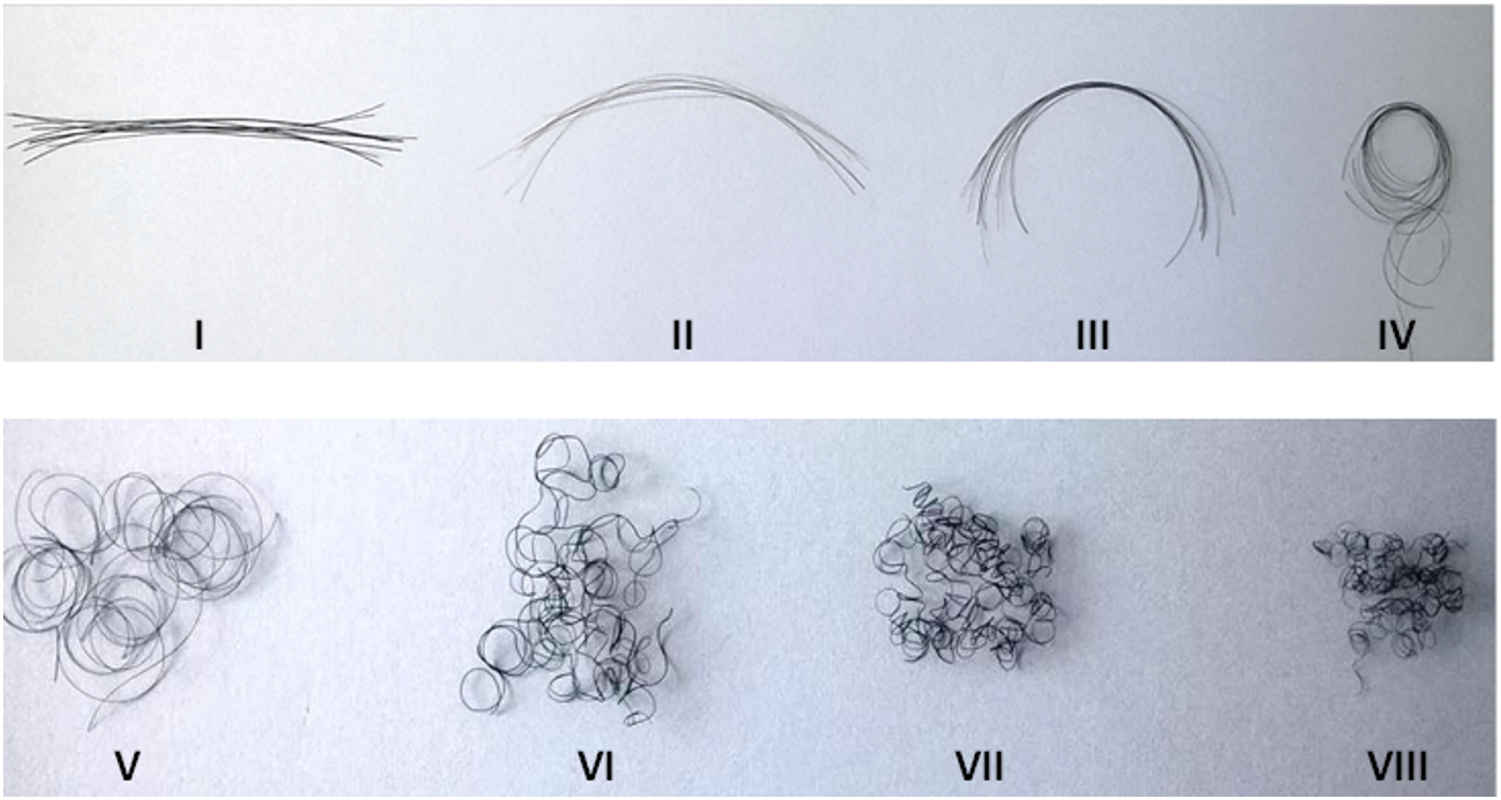
Demographics of sample population (n = 128) across 8 hair curl groups. Same number (10) of hair 6cm in length—each sample is representative of individuals geometrically classified into groups I–VIII using 3 measurements (curve diameter, curl meter, number of waves).

One evaluator also assessed (at a different time point) 10 hairs each for an additional 80 volunteers using the 6-group classification. These were observed to have similar intra-rater agreement [k = 0.754 (95% CI; 0.669–0.839)] as that reported for the 48 volunteer sample for this assessor. The overall distribution of classified hair curl groups corresponded with subjective racial classification, where Asian hair is usually straight, Caucasian hair is straight to curly and African hair was the curliest (Figs 1 and 2).

## Discussion

Self-declared racial groups showed significant overlap with each “race” falling into 3–4 of the 8-group and at least 2–3 of the 6-group classifications; thus confirming “race” as an unreliable descriptor of hair curl. Although representative hair samples (10 strands, one volunteer) from each geometric group demonstrate a gradual increase in hair curvature from group I to VIII; statistical analysis suggests that including the third step (‘counting the waves’) does not reliably distinguish curly hair phenotypes into 4 four groups (Group V versus VI and VII versus VIII) as previously suggested [26]. For all raters, classification of hair from the same volunteer fell into at least 2 (and occasionally 3) groups which may suggest observer error or heterogeneity of hair morphology even within a small area (0.562cm^2^) as previously reported [22,23]. The straight hair phenotype of Asian (Chinese and Indian) volunteers in our sample was grouped into group I to IV; the Indian participants likely contributed to the presence of hair curlier phenotypes. Participants who identified their race as Caucasian were classified in all hair curl groups except VII and VIII. The absence of participants who identified as mixed race in hair group VII was thought to be by chance. African hair ranged from curl group V to VIII. This was consistent with observations by Loussouarn et al [26]. and other workers [26,31,32].

Inter-rater agreement for the 8-group classification system was moderate [time 1 (k = 0.380) even though slightly improved (k = 0.455) at time 2]. This may reflect improved rater proficiency with the geometric classification.

The major challenge encountered with the 8-group classification system was the difficulty in handling the hair to count the number of waves for hair groups V to VIII. Further, the distinction of hair group V from VI and hair group VII from VIII was somewhat subjective. For instance, a strand could be a V or VI based on one extra or less wave. Eliminating this step (counting waves) from the classification resulted in improved inter and intra-rater agreement as seen in the kappa values of the 6-group classification (Tables 3 and 4).

The poorest inter-rater agreement values were for Hair curl groups I, VI and VIII (Table 1) for the 8-group and hair curl group 1 and 5 for the 6-group classification (Table 1); these could reflect the small number of subjects in these groups. This was partly because it is not possible to include equal numbers of all hair curl variation just by looking at the hair on the scalp. Further, the geometric classification at 2 time points sometimes fitted the hair into different groups, e.g. hair curl I were more in the 8-group than in the 6-group classification because both Chinese participants hair on repeat assessment ended up in the hair curl II group. The existence of the two extreme hair curl groups (I and VIII) is likely to reduce in future, [33] while groups III–V are predicted to increase because of increasing human migration and admixture. The distribution of hair curl groups according to race varied slightly when comparing the 8 and 6-group classifications (Fig 1a and 1b) with more group I hair in the 8 than the 6-group classification of the same volunteers. The most frequently occurring hair curl for each set of 10 strands was used as representative for the volunteer. This was done for all raters at time-1 and 2. In instances where no one hair curl was the most frequent in a set of 10 strands, the curliest hair group was used (i.e. considered more likely to be representative for the volunteer than straighter hair that could have straightened due to mechanical damage or extension). Temporary hair curl change as a result heat and/or cosmetic physical curling would not have survived the sample preparation steps. Chemically altered hair (straightened or curled) was excluded from the study. Further, hair samples were washed in between evaluators in order for the hair to regain its natural non-extended hair form. Lastly, the overall hair curl for each of the 48 volunteers was based on the most frequently occurring hair group of the 6 evaluations (i.e. from 60 hairs) made by the 3 raters each for the 8 and 6-group classifications.

## Conclusions

The 8 group geometric classification system presented by De La Mettri et al. and Loussouarn et al. is a useful contribution to the scientific use of hair for various applications but is unreliable. The 6-group geometric hair classification is quicker to perform, more reliable but still operator dependant. The poor kappa statistics for hair type 1 may reflect either small study samples or a need to further reduce the hair groups to 5 (and maybe even 4) but this needs confirmation in large studies. A valid and reliable tool for hair curl variation would be useful to investigate relationships between hair geometry and biochemistry and the potential influence on the reliable use of hair for drug incorporation, and disease predilection. However, a digital hair geometry tool would reduce operator error.

## Supporting information

S1 File Copy of data for kappa statistics. (XLSX)

S2 File Copy of data for ethnic and gender demographics. (XLSX)
